# The Role of Nurses' Uncertainty in Decision-Making Process of Pain Management in People with Dementia

**DOI:** 10.1155/2018/7281657

**Published:** 2018-08-01

**Authors:** Mohammad Rababa

**Affiliations:** Assistant Professor, Jordan University of Science and Technology, Jordan

## Abstract

Pain in people with dementia (PWD) is underassessed and undertreated. Treatment of pain in people with dementia goes awry because of poor assessment, poor treatment, and factors related to nursing decision-making skills. Several theoretical models addressed the role of nurses' critical thinking and decision-making skills in pain treatment, like the cognitive continuum theory (CCT) and the adaptive pain management (APT). Only the Response to Certainty of Pain (RCP) model was the first model to posit relationships between nurses' uncertainty, pain assessment, and patient outcomes. Gilmore-Bykovskyi and Bowers developed the RCP, which incorporates the concept of uncertainty and how it relates to the problem of unrelieved pain in PWD. The RCP model has the potential to provide good understanding of the problem of unrelieved pain in people with dementia. It also could help to develop a research study that brings comfort to an often neglected and vulnerable population.

## 1. Introduction

Pain is one of the most prevalent problems in the elderly; pain affects approximately 20% of the elderly population [1]. Chronic pain in patients aged 85 years or older is a common problem, occurring in 40-79% of individuals [2]. Fifty-six of community-dwelling older adults and 70% of nursing home residents have pain [3]. This high prevalence of pain among older adult patients is explained in part by physiological age-related changes and comorbid problems that happen in later life (Gibson and Lussier, 2012; [2, 4]). According to Achterberg et al. [5], elderly people are more prone to receive poor pain management.

Despite the high prevalence of pain among older adults, people with dementia (PWD) appear to be at great risk of poor treatment of pain because they have difficulty communicating their unmet comfort needs [6]. According to Husebo et al. [7], pain affects 50% to 80% of nursing home (NH) residents with dementia. Several studies have also consistently shown the high prevalence of poor assessment and treatment of pain in NH residents with dementia [8–10]. For example, it has been reported that PWD are prescribed significantly less scheduled analgesics as well as receiving less analgesics than cognitively intact older adults even with controlling for number of painful situations [5, 11].

Treatment of pain in PWD goes awry because of many reasons. However, in this paper three reasons are chosen and will be discussed separately. These reasons include (a) poor assessment, (b) poor treatment, or (c) factors relating to nurses' critical thinking and decision-making skills. The purpose of this paper is to provide a brief overview of evidence related to poor assessment and poor treatment of pain in PWD and then to describe in more detail nurses' critical thinking and decision-making, with a description and explanation of how the Response to Certainty of Pain (RCP) model [12] may help elucidate how nurse uncertainty regarding suspected pain drives assessment and predicts patient outcomes. The RCP model may help researchers to understand factors that predict poor pain management and may aid in the development of interventions to improve treatment.

### 1.1. Assessment

There cannot be effective treatment without accurate assessment of pain [13]. Nurse scientists need to give much more attention to developing assessment tools that could improve practice and eliminate barriers to optimal pain management [14]. A study done by Kovach et al. [15] identified the need to develop more critical ways to properly assess pain in PWD. It is important that healthcare professionals conduct more appropriate and accurate pain assessment in order to accurately identify pain and provide effective treatment using a range of comforting interventions [15, 16]. With a better understanding of the problem of pain, healthcare providers may be able to improve the quality of life for PWD. Over the past few years, scientists have made significant progress in this area by developing, evaluating, and introducing a variety of observational assessment tools used to detect pain in PWD [17]. However, pain is still unrelieved in PWD [13].

Because of the complexity of pain assessment in PWD and the time needed to assess pain in this population, nurses may spend a few hours rather than minutes to assess their patients' pain (Prkachin, Solomon, and Ross, 2007). Nurses may be reluctant to perform a thorough assessment. Instead, these nurses may overly simplify the assessment process by relying on the rapid data processing evident when using intuition to discern pain sufferers from others [18].

### 1.2. Treatment

Unrelieved pain in PWD could also be explained in part by poor treatment [19]. Nurses are often concerned about drug addiction and other consequences of analgesic use, such as respiratory depression [19]. Also, nurses may have both knowledge deficits and misconceptions regarding the use of analgesics and pain in PWD [20]. Nurses often struggle with understanding the nature of pain in PWD [21]. They cannot differentiate between the pure sensation of pain and other unpleasant emotions [21]. Even if nurses identify suspected pain in a PWD, determining pain intensity, duration, and location is far from a simple exercise [11]. According to a recent study, 30% of PWD residing in nursing homes report pain daily, but 25% of those NH residents with pain do not receive analgesics [22]. Even if analgesics are prescribed and available for PWD, nurses often fail to administer those medications or tend to administer only a portion of the prescribed amount [12]. Moreover, a recent study showed that nurses' analgesic choices are mainly determined by the ability of patients to self-report their pain. As a result, nonverbal patients like PWD have little effect on the choice of analgesics [23].

Pharmacological treatment is considered to be the first option for pain management [19]. However, nurses may start with nonpharmacological treatment to address pain in PWD and delay analgesics. The reason for this is that nurses may have misconceptions about behavioral symptoms of pain in PWD. Nurses may conceptualize these behavioral symptoms from a psychological perspective rather than physical perspective. As a result, nurses may not prioritize treatment of physical problems and try instead to treat the nonphysical underlying cause of behavioral changes, such as psychosocial causes [11, 21].

In addition, even though nurses use the pharmacological treatment to manage pain in PWD, they may use antipsychotic drugs instead of analgesics. The use of antipsychotic drugs is often associated with decreased level of functioning, unmet care needs, increased serious cardiovascular events, and decreased quality of life [24]. Furthermore, some nurses are not sure whether PWD truly have pain or just want to be medicated. Despite nurses' efforts to treat pain in PWD, they may feel frustrated with this group of older adults who have difficulty verbalizing their pain [25].

## 2. Critical Thinking and Decision-Making

The underestimation of pain assessments and the undertreatment of pain in PWD are well known. Little is known about how nurses think about and make their decision about pain assessment and management in PWD. The problem of pain in PWD may be explained partly by poor critical thinking and decision-making skills on the part of nurses. To make an accurate decision about pain management in PWD, nurses should clearly identify the behavioral indicators of pain, such as facial grimacing, withdrawal, restless behaviors, and negative vocalizations. However, nurses do not have a clear understanding of what the behavior indicators in PWD should look like even in those who could self-report their pain. Instead, nurses often think of autonomic nervous system changes in vital signs, evident in acute pain conditions, and observable physical changes as cues to pain in PWD who are having acute or chronic pain [26].

Nursing homes in the US have a high percentage of unlicensed nurses in relation to licensed nurses [27]. According to the same study, 80% to 90% of care in nursing homes is delivered by Certified Nursing Assistants (CNAs). CNAs often do not receive high quality education and clinical training. Furthermore, the problem of a lack of staffing in nursing homes overwhelms many CNAs and CNAs are not responsible for assessment or clinical decision-making [28]. Consistently, a recent study by Kovach et al. [16] showed that the majority of nursing staff working in nursing homes are Licensed Practical Nurses (LPNs) and associate-degree registered nurses (RNs). Those nurses may have knowledge deficit and poor experience, which make them unqualified to deal with the complexity of pain assessment in PWD and make appropriate decisions regarding pain treatment.

According to Kaasalainen et al. [11], nurses acknowledged that a major problem of pain management in PWD lies in the decision-making process they undergo until they get analgesics prescribed. Nurses believe that the way they interact with pain in PWD is mainly based on a trial and error approach. Nurses often became confused when they were uncertain regarding suspected pain in PWD.

To overcome uncertainty regarding suspected pain in PWD, it has been recommended that nurses identify a variety of behavioral indicators to make their decisions about pain assessment. These indicators are based on the nurses' understanding of the usual behaviors of PWD [11]. Unfortunately current pain assessment strategies rarely incorporate formalized decision-making processes. Nurses often look at pain symptoms in PWD as just a manifestation of a behavior change associated with the dementing illness. Regardless of how different PWD show pain, it is discussed as and responded to as a sign of a dementing illness. The current body of evidence suggests that nurses often do not have a clear understanding of what pain looks like in PWD, which then leads to a state of uncertainty. This uncertainty impacts the nurses' decision-making as regards pain treatment [11]. This paper will present theoretical and empirical evidence supporting the premise that nurses' uncertainty regarding suspected pain in PWD may explain inadequate assessment, which then leads to undertreatment of pain.

## 3. Background of Decision-Making Theory

### 3.1. Background of Field of Healthcare Decision-Making

Decision-making about treatment is an essential part of modern healthcare [29]. Dementia causes a gradual decline in decision-making ability. Therefore, PWD may be unable to make decisions related to their treatment [30]. Healthcare providers play a significant role in making decisions about different treatment options, based on information regarding risks and benefits [29]. In the case of dementia, the role of healthcare providers is even more significant. Many researchers have described how nurses learn, apply what they learned in clinical settings, derive new knowledge from clinical experience, and demonstrate their decision-making skills to translate theoretical knowledge into practice [30]. In the following paragraphs, this paper will provide an overview of some of the main decision-making models used in the field dementia of healthcare.

### 3.2. Overview of Core Nurse Decision-Making Models in Pain Management

The implementation of evidence-based interventions in healthcare settings has increased the importance of understanding the nursing decision-making process. A thorough review of the literature regarding research studies previously conducted among nursing home residents with dementia was completed in the following databases: CINAHL plus with full text, Medline, ERIC, Education Research Complete, and Psychology and Behavioral Sciences Collection. The search was limited between 2000 and 2017 using the following combination of search terms: “nursing home residents” “dementia”, “decision-making”, and “pain”. Articles relatedness to the focus of describing nurse decision-making models on pain treatment in NH residents with dementia was evaluated. The retrieved articles discussed only three main theoretical models including cognitive continuum theory (CCT) [31], adaptive pain management (APM) [32], Shields et al. [33] model, and RCP model [12]. 

*Cognitive Continuum Theory*. The CCT is a descriptive theory that explains the relationship between a decision-making task and an individual's cognition. According to it the individuals' cognition is a continuum ranging from intuition to analysis. Analysis has been defined as slow processing of data with high consciousness and high certainty. Intuition is defined as a quick processing of data with low consciousness and low certainty. Quasi-rationality is considered to be the central area in the cognitive continuum that moderates the interaction between the other two modes of cognition: intuition and analysis [31]. Also according to the CCT, there are two types of decision-making tasks including well-structured and ill-structured tasks. Well-structured tasks induce an analytical thought process through decomposing the tasks into many sections. Although this process takes time to resolve the task, it has a high degree of certainty. On the other hand, ill-structured tasks induce intuitive process through decomposing the task into a very few sections. Although this process does not take time to resolve the task, it has a low level of certainty [31].

While the CCT theory gives a very good explanation of the difference between analytical and intuitive processes needed to make decisions, the processes described are quite linear and simple and fail to accommodate the multifactorial or complex nature of pain in PWD. Although the analytical process is justifiable, it is complicated. Also, although intuitive process is quick and flexible, it is imprecise and irretraceable. Intuitive errors happen unexpectedly and are often hard to be identified (Dhami and Thomson, 2012). Also, the mode of cognition that the CCT uses lacks rationality and overly relies on intuition, which has been presented as the origin of irrationality or guessing. The complexity of nurses' decision-making process suggests that intuition is insufficient for nursing intervention. Furthermore, the model does not account for or capture uncertain or ambiguous thinking in the decision-making process [34]. Research using the CCT has involved small samples with weak designs and no research has examined the association between the nurses' decision-making certainty and assessment type with agitation and pain levels in PWD. 

*Adaptive Pain Management (APM).* The APM is a decision-making framework that aims to frequently review past and current information of patients to determine the best choice of treatment [32]. The APM demonstrates a decision-making process based on dynamic programming (DP) to create an adaptive treatment of pain. The APM has two main stages that end with DP formulation and solution process presentation. The first stage, prior to treatment, includes an evaluation of the characteristics of pain, related health parameters and medical history, and ends with the determination of a treatment regime. The second stage determines whether an adjustment in the treatment should be made or not and comes up with the final outcomes. The final decision in both stages is the on-hand treatment plans, which could be a combination of a number of different pharmacological or nonpharmacological interventions [32].

While the APM theory identifies the best choice of pain treatment in elderly patients and demonstrates a decision support system based on dynamic programming process, the process implies that nurse should continually adapt and readapt the treatment to the patient [32]. This continuous adaptation and readaptation of treatment rely on patient feedback which means that patients should be able to verbalize their unmet needs. Therefore, the model does not account for cognitive impairment and communication deficit in PWD [35]. Also, the model does not account for or capture uncertain or ambiguous thinking in the decision-making process. Research using the APM has involved small samples, with weak designs, and no research has examined the association between the nurses' decision-making certainty and assessment type with agitation and pain levels in PWD [35].

## 4. Uncertainty and Pain Assessment

Two theories have directly addressed the role of uncertainty in pain management in PWD. A model by Shields et al. [33] states that the decision-making process is a complex intellectual process of clinical reasoning or judgment that includes making explanations regarding what level of certainty nurses have in taking action. Action means the implementations the nurse decides or does not decide to do. Not taking action means not initiating an intervention. Both actions and nonactions are the result of the decision-making process, and both involve a process of making explanations [33]. While the work of Shields et al. posits that when recall of nurses' academic education and clinical experience, attitudes, values, beliefs, and culture to the situation may increase or decrease the level a nurse's level of certainty about pain in PWD, explanations within the model are not well developed and lack direct applicability to clinical situations of complex assessment of pain in PWD.

### 4.1. Introduction to the Response to Certainty of Pain (RCP) Model

The Response to Certainty of Pain (RCP) model by Gilmore-Bykovskyi and Bowers [12] illustrates two trajectories for nurses' assessment and management of pain for NH residents: one when the nurse is certain the patient is in pain and one when the nurse is uncertain whether the patient is in pain. When the nurse is certain that the patient is in pain, the trajectory moves in a timely fashion from suspected pain to treatment. When the nurse is uncertain, the patient is in pain the trajectory from suspected pain to treatment is delayed or never accomplished [12].

The uncertainty trajectory is often slowed down by a time-consuming assessment process based on trial and error. Certainty of pain is quickly confirmed by prompt treatment in the certainty trajectory. In the uncertainty trajectory, although various treatments may be tried, the nurse may never be certain that the patient was in pain because the path from suspected pain to treatment is more convoluted. Furthermore, in many cases the path never leads to treatment so there is no treatment outcome to confirm the presence of pain [12].

Gilmore-Bykovskyi and Bowers' model of how certainty and uncertainty create different trajectories for nurses' decision-making in pain management for nursing home patients could lay the foundation for much needed translational research to improve nursing practice for patients with dementia [12]. The RCP model demonstrates that the role of certainty would be a key nursing factor in future gerontological pain research. The RCP model is the first model to posit relationships between nurses' uncertainty, pain assessment, and patient outcomes of pain and agitation in PWD. The RCP model has been tested in a correlational study among a convenience sample of 78 NH residents with dementia and all study's hypotheses derived from the model were supported [2, 4].

### 4.2. Nurses' Certainty Trajectory

When nurses are certain regarding suspected pain they do a brief self-report assessment and provide timely pain management. According to the RCP model, nurses implement timely pain management if residents do not have dementia because their behaviors are highly suggestive of pain. In addition, nurses respond with a high level of certainty regarding suspected pain in patients with a short-term stay in the nursing home, even though nurses know that not all residents staying for short period of time are cognitively intact.

According to Gilmore-Bykovskyi and Bowers [12], nurses try to confirm suspected pain in older adult patients who do not have dementia by asking the patient for a self-report. In case of PWD, nurses become more certain regarding suspected pain and provide timely treatment if PWD show obvious pain indicators. When nurses feel a high level of certainty about pain in their PWD they are more likely to quickly validate their assessments by interviewing family members, contacting a physician, taking vital signs, or checking medical records and to confirm their certainty about the presence of pain.

### 4.3. Nurses' Uncertainty Trajectory

Nurses are uncertain regarding suspected pain in PWD for a number of reasons. The complexity of pain assessment in PWD does not enable nurses to suspect pain in their patients. Furthermore, the severity of dementia strongly affects the level of a nurse's certainty. As the severity of dementia increases, a nurse's level of certainty regarding suspected pain decreases [12]. Moreover, nurses may not suspect pain in PWD because of the inconsistency of their behavior and because pain may not be the primary cause of their behavior.

According to the RCP model, when nurses are uncertain regarding suspected pain in PWD, they do not confirm certainty and evaluate the effectiveness of the pain treatment given to PWD. Nurses do not try to ease their uncertainty by asking PWD if they are in pain, because PWD cannot verbalize their pain. Instead, nurses try to ease their uncertainty by conducting additional assessment to increase certainty and doing trial and error process. In this case, treatment is focused on reversing behavioral changes and consequently the treatment of pain may be delayed or not provided at all. Also, according to the model, nurses are consistently uncertain regarding suspected pain in elderly with long-term stays. Those elderly usually have many comorbid problems that make nurses unable to suspect pain because they believe pain generally is not anticipated in a long-term stay [2, 4]. In this situation, nurses also do further assessments and include additional decision-making processes. As a result, the treatment of pain may be delayed. Also, according to Gilmore-Bykovskyi and Bowers [12], nurses sometimes were not allowed by physicians and administrators to make their own decisions to select the type of pain assessment tools used for PWD. Actually, it could be a disadvantage of the RCP model that it puts the nurse in a central role to change poor pain management into good pain management, although in practice there might be other factors of influence beyond her reach.

### 4.4. Other Theoretical References

PWD might be unable to provide meaningful and clear verbal information about their pain experience. Therefore, they are unable to confirm nurses' certainty or uncertainty regarding suspected pain. As a result, nurses might refer to their interpretations of subjective and objective data of pain, evaluating behavioral indicators of pain. Pain cues are defined as changes in behaviors such as aggression and restlessness/agitation, sounds such as vocalization, or appearance such as facial expressions [36]. Recognizing these behavioral changes needs additional pain assessment and more decision-making steps. The nurses' ability to interpret these changes is limited, impeding them to make certain decisions about pain treatment (Parke, 1998).

Nurse also may be unable to discriminate between pain and dementia [11]. Nurses acknowledge that they are often uncertain about pain in PWD, as they are unsure whether behavioral changes are pain-related or dementia-related. In most cases, nurses believed these behavioral changes are caused by something other than pain. Indeed, the last thing that nurses think about when they care for PWD who show change in behaviors is pain. Consequently, nurses would try to manage these behavioral changes based on a pragmatic approach rather than theoretical considerations. As nurses often do not have a definitive diagnosis of pain in PWD, they struggle with treatment options and frequently question their decisions about chosen treatment of pain.

When nurses are uncertain regarding suspected pain in PWD, they tend to use their intuition. In this case, nurses not only cannot recognize the behavioral changes in PWD but also are unable to clearly translate their intuition into meaningful data, facilitating clinical decisions about pain [37]. For example, nurses may report that they just feel inside something is wrong because the patients are not being themselves. In this example, nurses' intuition is not enough to develop certainty regarding suspected pain in PWD [37]. When nurses use their intuition to assess pain in PWD, they are uncertain if the behavioral changes they observe represent pain.

In addition, nurses do not have clear understanding of decision-making processes and other relevant information. Understanding other relevant information includes medical diagnoses, pathophysiology, and the nurses' personal pain experiences. The decision-making process involves recognizing the PWD's specific pain cues, effectively assessing pain, administering analgesic in a timely manner, and evaluating the effectiveness of the intervention. However, nurses use a process of trial and error instead. This process involves administering nonpharmacological intervention and observing PWD's responses to these interventions or just looking for the reversal of behavioral changes [33].

### 4.5. Evidence That Relates to Uncertainty and Pain Treatment

Because healthcare providers are uncertain regarding suspected pain in PWD, PWD are prescribed significantly fewer analgesics [5]. For example, when physicians prescribe analgesics for PWD after hip fracture surgery, they put them at a dosage that is one-third of that prescribed for cognitively intact patients [5]. Furthermore, when physicians are uncertain about pain in PWD, they start with a very low dose of analgesics and then increase the dosage if a low dose does not work. However, physicians and nurses are not sure of whether and how much they should increase the dosage of analgesics. They may start to think that maybe it is not actually pain; maybe this is part of dementia, not pain [11].

Furthermore, even though physicians may prescribe analgesics for PWD, nurses may sometimes be uncertain about pain, and consequently PWD may not receive adequate pain treatment. A recent study by Kaasalainen et al. [11] found that nurses' uncertainty regarding suspected pain in PWD influenced the choice of opioid analgesics dose and contributed to poor treatment of pain. The same study found that nurses were not always certain about their ratings of pain, which were not significantly correlated with the administration of analgesics. Consequently, even though PWD have pain in the past, they do not always receive analgesics [11].

When nurses are uncertain about pain in PWD, they feel uncomfortable and hesitant to use analgesics to treat pain. They tend to administer analgesics only after all other nonpharmacological interventions have been tried and proven ineffective. According to a recent study, nurses who were uncertain whether a patient's behavior indicated pain were less likely to start pharmacological treatment than nurses who had higher levels of certainty [5]. Nurses who were uncertain about their patient's pain chose other pain interventions, delaying more effective pharmacological treatment. These other pain interventions include toileting, changing the room to fit resident preferences, providing acetaminophen, changing position, regulating the temperature of environment, reducing stimulation, doing outdoor activities, and providing food. Nurses and physicians are not willing to take the risks to treat pain with opioid analgesics. This reluctance to use analgesics results from their uncertainty regarding suspected pain in PWD [11].

## 5. Conclusion

PWD are at great risk of unrelieved pain due to poor assessment, inadequate management, or problems with decision-making process. Many decision-making theories including the CTT and APM have been developed on pain management recently. However, the RCP is the only theoretical framework that has examined the association between the nurses' decision-making certainty and assessment type with agitation and pain levels in PWD. The RCP provides an understanding of treatment decisions regarding pain in PWD. This article would suggest that nurses' uncertainty might explain poor treatment of pain in PWD.
